# The effect of selective serotonin re-uptake inhibitors on risk of type II diabetes mellitus and acute pancreatitis: a meta-analysis

**DOI:** 10.1042/BSR20180967

**Published:** 2018-10-05

**Authors:** Shun Yao, Jian Li, XiuDe Fan, QingQuan Liu, JianQi Lian

**Affiliations:** 1 School of Basic Medicine, The Fourth Military Medical University, Xi’an, Shaanxi, China; 2 Department of Infectious Diseases, First Affiliated Hospital of Xian Jiaotong University, Xian, Shaanxi, China; 3 Department of Endocrinology, TangDu Hospital, The Fourth Military Medical University, Xian, Shaanxi, China; 4 Department of Infectious Diseases, TangDu Hospital, The Fourth Military Medical University, Xian, Shaanxi, China

**Keywords:** acute pancreatitis, meta-analysis, selective serotonin re-uptake inhibitors, type II diabetic mellitus

## Abstract

To explore the effect of selective serotonin re-uptake inhibitors (SSRIs) on risk of type II diabetes mellitus (T2DM) and acute pancreatitis (AP), expecting to provide guidance for clinic. Literature was retrieved by searching Pubmed, Embase, Cochrane and Scopus and hand searching of reference lists of related articles. Stata 14.0 was utilized for processing and analysis, and adjusted odds ratios (aORs) were applied. Our study included 113898 T2DM patients and 284131 controls from nine studies and 17548 AP patients and 108108 controls from four studies. The pooled aORs of SSRIs on the risk of T2DM and AP were 1.38 (95% confidence interval (CI) = 1.24–1.54) and 1.26 (95% CI = 1.13–1.40), respectively. Study design, quality, ethnicity, follow-up, and sample size of patients were the resources of heterogeneity. Subgroup analysis showed that 2 weeks is a high-risk time for AP after SSRIs use, with 1.48-fold-times as much after it. This meta-analysis provides evidence of a significant positive association between SSRIs use and risks of T2DM or AP, and duration of 2 weeks of SSRIs use has higher risk of AP, which should be paid much attention to.

## Introduction

Type II diabetes mellitus (T2DM), an adult-onset disorder and characterized by insulin resistance, is increasing amongst children, adolescents, and young adults in recent years [1], and its projected worldwide prevalence is expected to reach 642 million by 2040 [2], which has been a national and world health issue. Acute pancreatitis (AP), an inflammatory disorder of the pancreas, whose annual incidence ranges from 13 to 45 per 100000 people [3], is the leading cause of admission to hospital for gastrointestinal disorders. In 2009, acute AP became the most frequent principal discharge diagnosis in gastrointestinal disease and hepatology in U.S.A. [4].

Selective serotonin re-uptake inhibitors (SSRIs), as a new second-generation antidepressant, has become a first-line medication for depression, account for their safer and better tolerance than other types of antidepressants [5]. The use of SSRIs is widespread, they make up approximately 62% of all antidepressants in the United States [6], including sertraline, paroxetine, fluoxetine, citalopram, and escitalopram. However, concern of the safety of SSRIs is growing in recent years. Several adverse effects of SSRIs have been reported, including bleeding risk [7], autistic offspring [8], fractures [9], and stroke [10], and so on. Some researchers reported that SSRIs may be associated with an increased incidence of AP [11–14] and T2DM [15–21], but with inconsistent opinion. Therefore, we explored SSRIs on the risk of them. In addition, although this meta-analysis of observational studies about antidepressants on the risk of T2DM have been published recently [22–24]; there was no detailed analysis of the relationship between SSRIs and T2DM. We further explore the relationship between SSRI and T2DM specifically on this basis.

## Materials and methods

### Literature search and including criteria

Systematic search by retrieving PubMed, Embase, Cochrane, and Scopus to obtain relevance articles through December 2017. The following keywords were used for searching: ‘serotonin re-uptake inhibitors’ or ‘SSRIs’ or ‘sertraline’ or ‘paroxetine’ or ‘fluoxetine’ or ‘fluvoxamine’ or ‘citalopram’ or ‘escitalopram’ and ‘diabetic mellitus’ and ‘acute pancreatitis’. Cited references of the retrieved articles and reviews were also checked. Reference lists were screened to expect or obtain new articles. This systematic review and meta-analysis are reported in accordance with the Preferred Items for Systematic Reviews and Meta-analysis (PRISMA) Statement [25]. We included any study that met all of the following inclusion criteria: (i) published literatures in English; (ii) independent case–control studies or cohort studies; (iii) the outcomes of interest were T2DM and AP; (iv) the original studies must provide the number of each group or the odds ratios (ORs), relative risks (RRs), or hazard ratios (HRs) of T2DM or AP. Excluded criteria: (i) duplicated data; (ii) the original data could not be extracted; (iii) animal experiment, basic research, cross-sectional studies, and no control group of patients; (iv) review, letter, case report, and no related study; (v) non-English language publication.

### Data extraction and assessment of methodological quality

The extracted data consisted of the following items: the first author’s name, publication year, population (ethnicity), methods, study design, matching criteria, sex, age (years), total number of cases and controls, adjusted OR or RR or HR estimates, and the corresponding 95% confidence interval (CI) for SSRIs use and adjusted confounding variables. Quality of studies was evaluated by the Newcastle–Ottawa scale, as recommended by the Cochrane Non-Randomized Studies Methods [26], a total score of 6 or less was considered low quality and 7–9 was deemed high quality.

### Statistical analysis

The end points included AP and T2DM. Meta-analysis was performed to calculate pooled ORs with 95% CIs by using Stata 14.0. We assumed there was similarity amongst OR, RR, and HR, because the rates of AP and diabetes mellitus events were less than 20% [27]. Heterogeneity amongst studies was assessed by *I^2^* statistic, *P*<0.10 and *I^2^* > 50% indicated evidence of heterogeneity [28]. Random-effects model [29] was used to estimate the pooled adjusted OR (aOR). The OR and corresponding 95% CI were utilized to assess the associations. For the risk of T2DM with taking SSRIs, subgroups analysis about study design, methodological quality, ethnicity, follow-up, and sample of patients were conducted to explore the source of heterogeneity. And for the risk of AP, subgroups analyses about different durations of SSRIs and ethnicity were performed to further explore clinical relationship between SSRIs use and AP. Since the duration of SSRIs varied across studies, therefore, the shorter duration of SSRIs use was defined as a duration of exposure of ≤14 days, and the longer duration of SSRIs exposure was defined as a cumulative duration of exposure of 14 days to 1 year. Sensitive analysis was implemented by excluding heavy-weight studies. Funnel plot and Egger’s test were carried out to explore publication bias, the *P*-value of Egger’s test <0.05 was considered significant [30].

## Results

### Selection and characteristics of included studies

**Figure 1:**
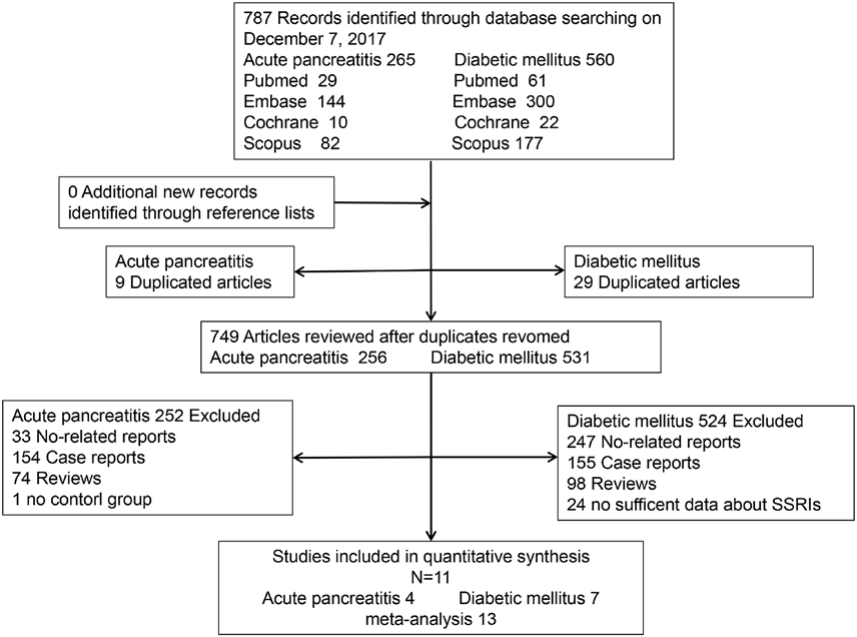
Flowchart of the literature search

The systematic search of PubMed, Embase, Cochrane, and Scopus provided a total of 787 citations, including 265 papers about AP and 522 papers about diabetes mellitus. After adjusting for duplicates and screening initial titles and abstracts, 749 were excluded. Twenty-six studies were potentially relevant studies, of which fifteen trials were excluded according to the exclusion criteria. Finally, eleven studies involved 17548 AP patients and 113898 T2DM patients were pooled for meta-analysis [11–21], including four nest case–control studies about AP [11–14], three cohort studies, and four nest case–control studies about T2DM [15–21]. In particular, one study reported results from three separate studies (Pan et al. [20] HPFS, NHS and NHSⅡ), which was analyzed separately in the meta-analysis, so nine studies about T2DM were included actually. No additional new articles were identified by screening references. A flow diagram of the study selection process was shown in Figure 1. All included studies were from eight countries or regions representing North America, Europe, and Asia. Duration of follow-up was from 2 to 12 years. And high-quality studies accounted for 63.6%. Characteristics of included studies were shown in Table 1.

**Table 1:** Characteristics of studies included in the meta-analysis

Study and year	Country	Design	Duration of follow-up (years)	Disease	Cases/controls	Adjustment variables	Quality of studies
Lancashire, 2003 [14]	U.K.	Nested case–control	9	AP	3673/11010	Age, sex, general practice, previous history of illness, height, weight, smoking habits and alcohol consumption, medication use (gastrointestinal system, cardiovascular system, central nervous system, anti-infective agents, immunosuppressants)	8
Lin, 2017 [11]	China	Nested case–control	13	AP	4631/4631	Age, sex, SSRIs use frequency, other antipressant drugs, comorbidities (alcohol-related diseases, biliary stones, cardiovascular diseases, chronic kidney disease, chronic obstructive pulmonary disease, diabetes mellitus (DM), hepatitis B and C, hyperparathyroidism, and hypertriglyceridemia)	8
Ljung, 2012 [13]	Sweden	Nested case–control	2	AP	6161/61637	Age, sex, history of excessive alcohol consumption or disease related to alcohol, drugs used to treat alcohol dependence, chronic obstructive lung disease, ischemic heart disease, antiobesity drugs, diabetes, antidiabetic medication, opioid drug use, educational level, marital status	8
Nørgaard, 2007 [12]	Denmark	Nested case–control	12	AP	3083/30830	Age, sex, SSRIs use frequency, other non-SSRIs antidepressant drugs, present use of other medicines (e.g. glucocorticoids, NSAIDs, antiepileptic, azathioprine), gallstone diseases, alcohol-related diseases, IBD	6
Andersohn, 2009 [15]	U.K.	Nested case-control	15	T2DM	2243/8963	BMI, hyperlipidemia, hypertension, smoking history, recent use of β-blockers, thiazides, phenytoin, antipsychotics, glucocorticoids, carbamazepine, valproate, lithium	9
Jerrell, 2009 [16]	U.S.A.	Cohort	9	T2DM	11970/4500	Age, gender, ethnicity	6
Khoza, 2012 [17]	U.S.A.	Cohort	7	T2DM	2937/9163	Age, gender, race, medication adherence, number of concomitant diabetogenic, medications, chronic disease, treatment duration	7
Kisely, 2009 [18]	Canada	Nested case–control	5	T2DM	608/607	Age, gender, use of psychotropic drugs: high-and low-potency conventional neuroleptics, olanzapine, quetiapine, risperidone, SSRIs, venlafaxine, amitryptiline, lithium, and other mood stabilizers	7
Kivimaki, 2010 [19]	Finland	Cohort	4	T2DM	851/4234	Hypertension, coronary heart disease, cerebrovascular disease, cancer	8
Pan, 2012 (HPFS) [20]	U.S.A.	Cohort	16	T2DM	1287/29411	Age, ethnicity, marital status, smoking status, alcohol intake, multivitamin and aspirin use, physical activity, metabolic equivalent, DM, BMI	8
Pan, 2012 (NHS I) [20]	U.S.A.	Cohort	12	T2DM	3514/57655	Mental health index	6
Pan 2012 (NHS II) [20]	U.S.A.	Cohort	12	T2DM	1840/68257	Menopausal status, hormone use, oral contraceptive	6
Wu 2014 [21]	China	Nested case–control	12	T2DM	47885/95770	Hyperlipidemia, presence of psychotics illnesses, use of other medications	7

BMI, body mass index; NSAID, nonsteroidal antiinflammatory drugs; IBD, inflammatory bowel disease.

### SSRIs and the risk of T2DM

**Figure 2:**
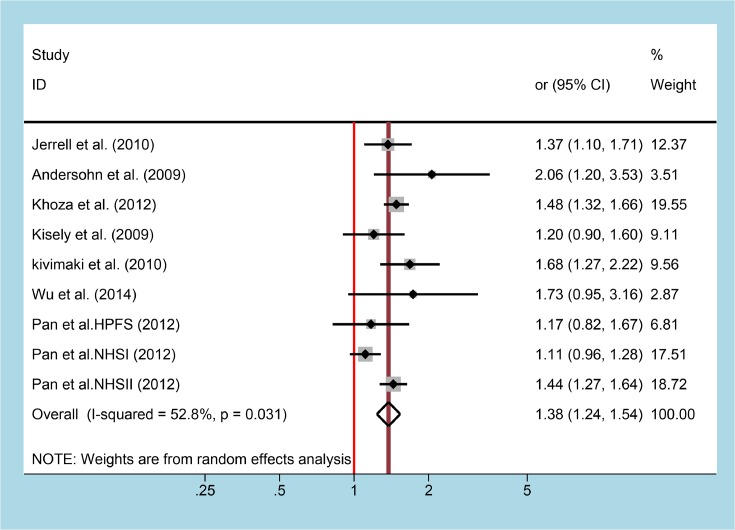
Forest plot for association between SSRIs use and risk of T2DM

**Figure 3:**
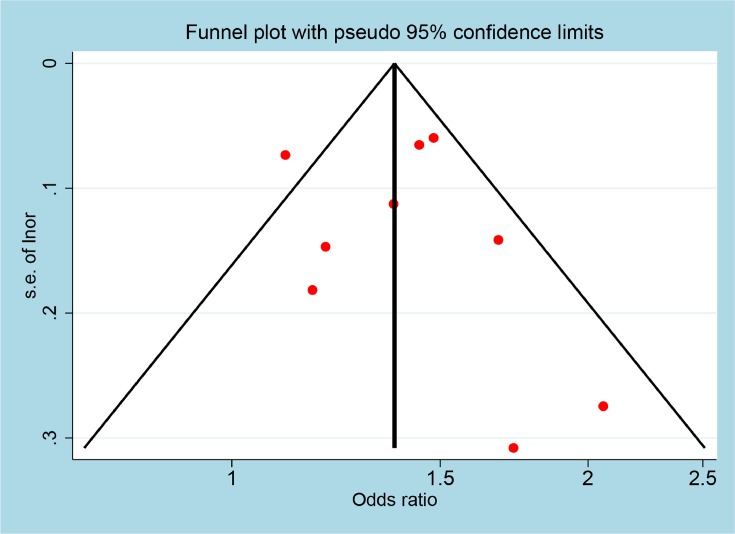
Funnel plot for association between SSRIs use and risk of T2DM

As shown in Figure 2, nine studies provided data about risk of T2DM with taking SSRIs, the pooled multivariate-aOR was 1.38 (95% CI = 1.24–1.54) with low heterogeneity (*P*=0.031, *I^2^* = 52.8%). Funnel plot was shown in Figure 3, Egger’s test (*P*=0.666) suggested that there was no publication bias. Sensitive analysis exhibited that our result was stable (OR = 1.34 (1.15–1.57)), without change significantly by excluding Khoza et al. [17] and Pan et al. [20] NHSII.

### SSRIs and risk of AP

**Figure 4:**
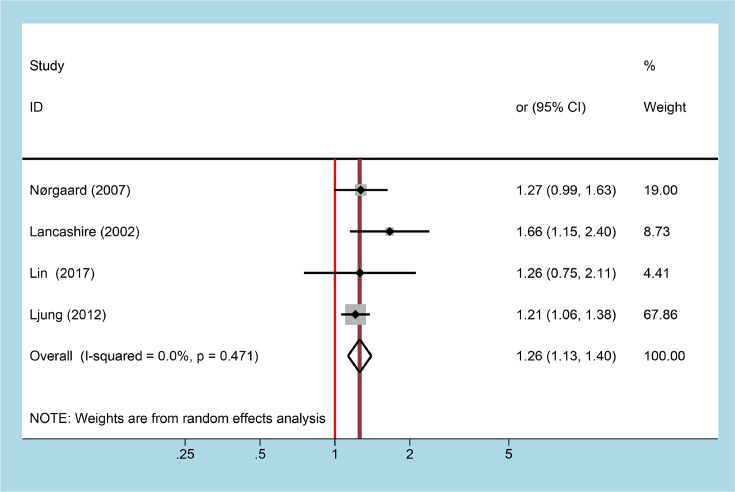
Forest plot for association between SSRIs use and risk of AP

Four studies providing data about risk of AP with taking SSRIs, the multivariate-adjusted pooled OR was 1.26 (95% CI = 1.13–1.40) with no heterogeneity (*P*=0.471, *I^2^* = 0.0%, Figure 4). Funnel plot could not be conducted because of limiting to small studies, Egger’s test (*P*=0.337) suggested no publication bias. Excluding Ljung et al. [13] to reanalyze results, the pooled estimate did not change significantly (OR = 1.26 (1.13–1.40)).

### Subgroup analysis

On assessment of the risk of T2DM with taking SSRIs, subgroup analysis (Table 2) about study design showed that significant differences appeared in both case–control studies (OR = 1.54, 95% CI = 1.22–1.95) and cohort studies (OR = 1.33, 95% CI = 1.17–1.51), but significant heterogeneity was only observed for cohort studies (*P*=0.025, *I^2^* = 64.1%). In subgroup analysis by methodologic quality, we discovered there was a significant result (OR = 1.45, 95% CI = 1.31–1.59) with no heterogeneity (*P*=0.370, *I^2^* = 7.6%) in high-quality studies (≥7 score), but this difference did not appear in low-quality studies (<7 score) (OR = 1.27, 95% CI = 0.98–1.64) with increased heterogeneity (*P*=0.008, *I^2^* = 85.8%). Outcomes of ethnicity exhibited that SSRIs could increase the risk of T2DM in both European (OR = 1.75, 95% CI = 1.37–2.24) and American (OR = 1.32, 95% CI = 1.18–1.47), but not Asian (OR = 1.73, 95% CI = 0.95–3.16). To explore further the resource of heterogeneity, subgroups analyses about follow-up and sample size of patients were also conducted, and all of the results suggested that pooled estimates were significant. And heterogeneity decreased obviously when follow-up <10 years and sample size of patients <2000. To explore the clinical relationship between SSRIs use and AP, we evaluated ethnicity difference and time-point of AP appearing after SSRIs use. The result indicated that SSRIs indeed could increase the risk of AP of European (OR = 1.28, 95% CI = 1.11–1.47), but may not of Asians (OR = 1.26, 95% CI = 0.75–2.11). What was more, shorter duration of SSRIs use was more strongly associated with AP (OR = 1.85, 95% CI = 1.27–2.69) than longer duration (OR = 1.25, 95% CI = 1.25–1.38).

**Table 2:** Subgroup analysis of SSRIs use and risk of T2DM and AP

Group	Subgroups	Number of studies	OR (95% CI)	*P*-value	*I^2^* (%)
**T2DM**					
Study design	Case–control	4	1.54 (1.22–1.95)	0.213	33.3
	Cohort	5	1.33 (1.17–1.51)	0.025	64.1
Study quality	>7 score	7	1.45 (1.31–1.59)	0.37	7.6
	<7 score	2	1.27 (0.98–1.64)	0.008	85.8
Ethnicity	European	2	1.75 (1.37–2.24)	0.509	0
	American	6	1.32 (1.18–1.47)	0.038	57.5
	Asian	1	1.73 (0.95–3.16)	-	-
Follow-up (years)	>10	5	1.34 (1.11–1.63)	0.025	64.0
	<10	4	1.44 (1.32–1.59)	0.372	4.1
Sample size of patients	>2000	5	1.40 (1.16–1.67)	0.014	68.2
	<2000	4	1.40 (1.23–1.60)	0.272	23.2
**AP**					
Ethnicity	European	3	1.28 (1.11–1.47)	0.283	20.7
	Asian	1	1.26 (0.75–2.11)	-	-
Duration of SSRIs use	≤14 days	2	1.85 (1.27–2.69)	0.374	0
	14 days to 1 year	3	1.25 (1.14–1.38)	0.523	0

## Discussion

Our study included nine observational studies to explore the effect of SSRIs on the risk of T2DM, the pooled adjusted OR showed that the risk increased 1.38 times with trying to calibrate as many other impact factors as possible, such as depression, hypertension and obesity, and so on. In addition, four studies in our study have for the first time identified evidence linking use of SSRIs to AP [11–14]. Remarkably, there was a significantly higher risk of AP with SSRIs, with approximately 1.26 times. Sensitive analysis and publication bias test showed that our results of T2DM and AP were both reliable.

A case–control study of Anderson is included in our meta-analysis. In this large observational study, which included more than 160000 patients with depression treated with antidepressants, the long-term use of antidepressants in moderate to high doses in patients with diabetes increased the risk of 84%. This risk includes both tricyclic antidepressants and SSRIs. An average of 3.2 years of continuous use of antidepressants increased the risk by 2.60 times of diabetes (95% CI = 1.37–4.94) [15]. A 10-year follow-up study showed that long-term use of antidepressants was associated with a significant increase in risk of T2DM, compared with the placebo group (OR = 2.34) [31,32].

Following are the possible mechanisms of diabetes caused by SSRIs. Boura-Halfon et al. have shown that SSRIs are potential inducers of insulin resistance, and its role may be to act as a direct inhibitor of the insulin signaling cascade in β-cells [33]. The mechanism of short-term inhibition of insulin by SSRIs may involve activating IRS-2 (insulin receptor substrate proteins-2) kinase, such as GSK 3 β, and promoting the phosphorylation of IRS-2 at some inhibitory Ser sites. He also found that GSK 3 β was a key factor in inhibition, including inhibition of protein function of IRS-2 and inhibition of GSIS (glucose-stimulated insulin secretion) [33]. On the other hand, by biochemical analysis and electrophysiological analysis, Paulmann et al. [34] found that 5-HT regulates insulin secretion by serotonylation of GTPases within pancreatic β-cells, and SSRIs can block this intracellular process, resulting in inhibition of insulin secretion in β-cells. In general, SSRIs can promote β-cell apoptosis, inhibit insulin secretion, and accelerate the transition of insulin resistance to dominant diabetes.

The resources of heterogeneity (*P*=0.031, *I^2^* = 52.8%) of pooled estimates of risk of T2DM with SSRIs taking was explored by subgroup analysis. We discovered that heterogeneity appeared obviously decreased when only nest case–control studies or high-quality studies were included. In addition, the similar results also appeared when follow-up <10 years or sample size of patients <2000. These results suggested that we not only found the resources of heterogeneity, but also knew that sample size had no impact on final results.

To further explore the clinical relationship between SSRIs use and risk of AP and provide help for clinic, we implemented subgroup analysis about ethnicity and time-point of AP appearing after SSRIs use. SSRIs use could increase the risk of AP of Europeans (OR = 1.28, 95% CI = 1.11–1.47), but may not of Asians (OR = 1.26, 95% CI = 0.75–2.11). But because of small studies included, we could not be sure that there exists ethnicity difference. As to time-point of AP appearing, we discovered that SSRIs use could increase the risk of AP strongly when ≤14 days (OR = 1.85, 95% CI = 1.27–2.69), with 1.48-fold-times of 14 days to 1 year, which we should pay great attention to risk of AP in 2 weeks after taking SSRIs.

Unfortunately, there has been no specific study to confirm the exact relationship between the occurrence of AP and the application of SSRIs. However, we can speculate that there may be some relationships amongst SSRIs, DM, and AP. In a cohort study of 337067 patients with T2DM, Noel et al. [35] showed that the risk of pancreatitis in T2DM group was 2.83 times (95% CI = 2.61–3.06) higher than that in non-diabetic patients. The risk of biliary tract disease was 1.91 times higher than that in non-diabetic group (95% CI = 1.84–1.99). There are also studies that followed up for 8 years, and they found that the incidence of AP in diabetics and non-diabetics was 2.98 and 1.68/1000, respectively. A covariable adjusted risk ratio is 1.53 (95% CI = 1.49–1.58) [36]. Hyperglycemia can induce oxidative stress in various tissues of the body. Chronic hyperglycemia has been shown to cause mitochondrial oxidative stress, which leads to increased production of reactive oxygen species (ROC) and lipid peroxidation [37]. What is more, ROC plays a key role in the pathogenesis of AP. [38]. With the progress of the disease, fibrosis in the inter-lobar septa is increasing. In the late stages of the disease, exocrine parenchyma is almost entirely replaced by fibrous tissue [39], with a mean β-cell deficit of 40–50% in patients with overt diabetes [40]. In addition, study found that 16.6% (*n*=1540) of patients with type II diabetes had elevated levels of serum lipase (1.2% of which were more than three times higher). Amylase levels increased in 11.8% (*n*=1094) of the patients (0.2% of them more than three times higher) [41]. Therefore, in the face of diabetic patients, clinicians also need to consider the possibility of accompanied pancreatitis.

Our study concluded that SSRIs may increase the risk of T2DM and AP indeed with giving much more specific explanations about the relationship between SSRIs and T2DM; and for the first time identified evidence linking use of SSRIs to AP. However, there were some limits existing that we could not avoid. First, despite aORs performed, we failed to explain the source of heterogeneity amongst subgroup analysis completely, because not all the adjusted variables were exactly the same for each study. Second, subgroup analysis about type of SSRIs was not conducted limited to small number of studies, in this case, we explored other possible variability. Third, we could not obtain sufficient data to carry out subgroup analysis, such as dose, duration. Fourth, we only included articles with English language, so relevant studies in non-English might be missed, which lead to publication bias in a certain degree. Finally, only observational studies were included in the analysis, a cause-and-effect relationship could not be established.

In conclusion, SSRIs could increase the risk of T2DM and AP, approximately 1.38-fold and 1.26-fold, respectively. In addition, the risk of AP was higher in the duration of ≤14 days with SSRIs use. Therefore, we should pay more attention to those taking SSRIs to prevent T2DM and AP. Although not so many studies have been included, sample size is large enough to support our conclusion. In the future, we hope that some high-quality studies about types, specific duration, intervals, and dose of SSRIs which trigger T2DM and AP are needed.

## Abbreviations

aOR: adjusted odds ratio
AP: acute pancreatitis
CI: confidence interval
GSK: glycogen synthase kinase
HPFS: Health Professionals Follow-up Study
HR: hazard ratio
5-HT: 5-hydroxytryptamine
IRS-2: insulin receptor substrate protein-2
NHSI: Nurses’ Health Study I
NHSII: Nurses’ Health Study II
OR: odds ratio
ROC: reactive oxygen species
RR: relative risk
SSRI: selective serotonin re-uptake inhibitor
T2DM: type II diabetes mellitus

## References

[1] Chen L., Magliano D.J. and Zimmet P.Z. (2011) The worldwide epidemiology of type 2 diabetes mellitus–present and future perspectives. Nat. Rev. Endocrinol. 8, 228–236 10.1038/nrendo.2011.183 22064493

[2] Reusch J.E. and Manson J.E. (2017) Management of Type 2 diabetes in 2017: getting to goal. JAMA 317, 1015–1016 10.1001/jama.2017.0241 28249081 PMC5894353

[3] Yadav D. and Lowenfels A.B. (2013) The epidemiology of pancreatitis and pancreatic cancer. Gastroenterology 144, 1252–1261 10.1053/j.gastro.2013.01.068 23622135 PMC3662544

[4] Peery A.F., Dellon E.S., Lund J., Crockett S.D., McGowan C.E., Bulsiewicz W.J. (2012) Burden of gastrointestinal disease in the United States: 2012 update. Gastroenterology 143, 1179–1187.e1-3 10.1053/j.gastro.2012.08.002 22885331 PMC3480553

[5] Karasu TB GAMA (2009) American Psychiatric Association practice guideline for the treatment of patients with major depressive disorder (second edition). http://www.psychiatryonline.com/pracGuide/loadGuidelinePdf.aspx?file=MDD2e_05-15-06

[6] Pirraglia P.A., Stafford R.S. and Singer D.E. (2003) Trends in prescribing of selective serotonin reuptake inhibitors and other newer antidepressant agents in adult primary care. Prim. Care Companion J. Clin. Psychiatry 5, 153–157 10.4088/PCC.v05n0402 15213776 PMC419384

[7] Laporte S., Chapelle C., Caillet P., Beyens M.N., Bellet F., Delavenne X. (2017) Bleeding risk under selective serotonin reuptake inhibitor (SSRI) antidepressants: a meta-analysis of observational studies. Pharmacol. Res. 118, 19–32 10.1016/j.phrs.2016.08.017 27521835

[8] Andalib S., Emamhadi M.R., Yousefzadeh-Chabok S., Shakouri S.K., Hoilund-Carlsen P.F., Vafaee M.S. (2017) Maternal SSRI exposure increases the risk of autistic offspring: a meta-analysis and systematic review. Eur. Psychiatry 45, 161–166 10.1016/j.eurpsy.2017.06.001 28917161

[9] Wu Q., Bencaz A.F., Hentz J.G. and Crowell M.D. (2012) Selective serotonin reuptake inhibitor treatment and risk of fractures: a meta-analysis of cohort and case-control studies. Osteoporos. Int. 23, 365–375 10.1007/s00198-011-1778-8 21904950

[10] Shin D., Oh Y.H., Eom C.S. and Park S.M. (2014) Use of selective serotonin reuptake inhibitors and risk of stroke: a systematic review and meta-analysis. J. Neurol. 261, 686–695 24477492 10.1007/s00415-014-7251-9

[11] Lin H.F., Liao K.F., Chang C.M., Lin C.L. and Lai S.W. (2017) Association of use of selective serotonin reuptake inhibitors with risk of acute pancreatitis: a case-control study in Taiwan. Eur. J. Clin. Pharmacol. 73, 1615–1621 10.1007/s00228-017-2328-x 28856398

[12] Nørgaard M., Jacobsen J., Gasse C., Pedersen L., Mortensen P.B. and Sørensen H.T. (2007) Selective serotonin reuptake inhibitors and risk of acute pancreatitis. J. Clin. Psychopharmacol. 27, 259–262 10.1097/JCP.0b013e318058a9c3 17502772

[13] Ljung R., Rück C., Mattsson F., Bexelius T.S., Lagergren J. and Lindblad M. (2012) Selective serotonin reuptake inhibitors and the risk of acute pancreatitis. J. Clin. Psychopharmacol. 32, 336–340 10.1097/JCP.0b013e318253d71a 22544014

[14] Lancashire R.J., Cheng K. and Langman M.J. (2003) Discrepancies between population-based data and adverse reaction reports in assessing drugs as causes of acute pancreatitis. Aliment. Pharmacol. Ther. 17, 887–893 10.1046/j.1365-2036.2003.01485.x 12656691

[15] Andersohn F., Schade R., Suissa S. and Garbe E. (2009) Long-term use of antidepressants for depressive disorders and the risk of diabetes mellitus. Am. J. Psychiatry 166, 591–598 10.1176/appi.ajp.2008.08071065 19339356

[16] Jerrell J.M. (2010) Neuroendocrine-related adverse events associated with antidepressant treatment in children and adolescents. CNS Neurosci. Ther. 16, 83–90 10.1111/j.1755-5949.2009.00106.x 19769598 PMC6493834

[17] Khoza S., Barner J.C., Bohman T.M., Rascati K., Lawson K. and Wilson J.P. (2012) Use of antidepressant agents and the risk of type 2 diabetes. Eur. J. Clin. Pharmacol. 68, 1295–1302 10.1007/s00228-011-1168-3 22120432

[18] Kisely S., Cox M., Campbell L.A., Cooke C., Gardner D. (2009) An epidemiologic study of psychotropic medication and obesity-related chronic illnesses in older psychiatric patients. Can. J. Psychiatry 54, 269–274 10.1177/070674370905400408 19321033

[19] Kivimaki M., Hamer M., Batty G.D., Geddes J.R., Tabak A.G., Pentti J. (2010) Antidepressant medication use, weight gain, and risk of Type 2 diabetes: a population-based study. Diabetes Care 33, 2611–2616 10.2337/dc10-1187 20823343 PMC2992199

[20] Pan A., Sun Q., Okereke O.I., Rexrode K.M., Rubin R.R., Lucas M. (2012) Use of antidepressant medication and risk of type 2 diabetes: results from three cohorts of US adults. Diabetologia 55, 63–72 10.1007/s00125-011-2268-4 21811871 PMC3229672

[21] Wu C.S., Gau S.S. and Lai M.S. (2014) Long-term antidepressant use and the risk of type 2 diabetes mellitus: a population-based, nested case-control study in Taiwan. J. Clin. Psychiatry 75, 31–38 10.4088/JCP.13m08421 24502860

[22] Bhattacharjee S., Bhattacharya R., Kelley G.A. and Sambamoorthi U. (2013) Antidepressant use and new-onset diabetes: a systematic review and meta-analysis. Diabetes Metab. Res. Rev. 29, 273–284 10.1002/dmrr.2393 23390036 PMC4888867

[23] Yoon J.M., Cho E.G., Lee H.K. and Park S.M. (2013) Antidepressant use and diabetes mellitus risk: a meta-analysis. Korean J. Fam. Med. 34, 228–240 10.4082/kjfm.2013.34.4.228 23904952 PMC3726790

[24] Salvi V., Grua I., Cerveri G., Mencacci C. and Barone-Adesi F. (2017) The risk of new-onset diabetes in antidepressant users – a systematic review and meta-analysis. PLoS ONE 12, e0182088 10.1371/journal.pone.0182088 28759599 PMC5536271

[25] Moher D., Liberati A., Tetzlaff J., Altman D.G. and (2009) Preferred reporting items for systematic reviews and meta-analyses: the PRISMA statement. Ann. Intern. Med. 151, 264–269, 10.7326/0003-4819-151-4-200908180-00135 19622511

[26] Wells G., Shea B., O’Connell D. and Tugwell P. (2014) The Newcastle–Ottawa scale (NOS) for assessing the quality of non-randomized studies in meta-analysis. Appl. Eng. Agric. 18, 727–734

[27] Davies H.T., Crombie I.K. and Tavakoli M. (1998) When can odds ratios mislead? BMJ 316, 989–991 10.1136/bmj.316.7136.989 9550961 PMC1112884

[28] Ioannidis J.P.A., Patsopoulos N.A. and Evangelou E. (2007) Heterogeneity in meta-analyses of genome-wide association investigations. PLoS ONE 2, e841 10.1371/journal.pone.0000841 17786212 PMC1950790

[29] DerSimonian R. and Kacker R. (2007) Random-effects model for meta-analysis of clinical trials: an update. Contemp. Clin. Trials 28, 105–114 10.1016/j.cct.2006.04.004 16807131

[30] Egger M., Smith G.D., Schneider M. and Minder C. (1997) Bias in meta-analysis detected by a simple, graphical test. BMJ 315, 629–634 10.1136/bmj.315.7109.629 9310563 PMC2127453

[31] Rubin R.R., Ma Y., Marrero D.G., Peyrot M., Barrett-Connor E.L., Kahn S.E. (2008) Elevated depression symptoms, antidepressant medicine use, and risk of developing diabetes during the diabetes prevention program. Diabetes Care 31, 420–426 10.2337/dc07-1827 18071002 PMC2373986

[32] Rubin R.R., Gaussoin S.A., Peyrot M., DiLillo V., Miller K., Wadden T.A. (2010) Cardiovascular disease risk factors, depression symptoms and antidepressant medicine use in the Look AHEAD (Action for Health in Diabetes) clinical trial of weight loss in diabetes. Diabetologia 53, 1581–1589 10.1007/s00125-010-1765-1 20422396 PMC3099396

[33] Isaac R., Boura-Halfon S., Gurevitch D., Shainskaya A., Levkovitz Y. and Zick Y. (2018) Selective serotonin reuptake inhibitors (SSRIs) inhibit insulin secretion and action in pancreatic beta cells. J. Biol. Chem. 293, 4577–4578 10.1074/jbc.AAC118.002476 29572328 PMC5868260

[34] Paulmann N., Grohmann M., Voigt J.P., Bert B., Vowinckel J., Bader M. (2009) Intracellular serotonin modulates insulin secretion from pancreatic beta-cells by protein serotonylation. PLoS Biol. 7, e1000229 10.1371/journal.pbio.1000229 19859528 PMC2760755

[35] Noel R.A., Braun D.K., Patterson R.E. and Bloomgren G.L. (2009) Increased risk of acute pancreatitis and biliary disease observed in patients with type 2 diabetes: a retrospective cohort study. Diabetes Care 32, 834–838 10.2337/dc08-1755 19208917 PMC2671118

[36] Shen H.N., Chang Y.H., Chen H.F., Lu C.L. and Li C.Y. (2012) Increased risk of severe acute pancreatitis in patients with diabetes. Diabetes Med. 29, 1419–1424 10.1111/j.1464-5491.2012.03680.x 22506974

[37] Kamboj S.S. and Sandhir R. (2011) Protective effect of N-acetylcysteine supplementation on mitochondrial oxidative stress and mitochondrial enzymes in cerebral cortex of streptozotocin-treated diabetic rats. Mitochondrion 11, 214–222 10.1016/j.mito.2010.09.014 21059408

[38] Halangk W. and Lerch M.M. (2004) Early events in acute pancreatitis. Gastroenterol. Clin. 33, 717–731 10.1016/j.gtc.2004.07.009 15528014

[39] Schrader H., Menge B.A., Schneider S., Belyaev O., Tannapfel A., Uhl W. (2009) Reduced pancreatic volume and beta-cell area in patients with chronic pancreatitis. Gastroenterology 136, 513–522 10.1053/j.gastro.2008.10.083 19041312

[40] Schrader H., Menge B.A., Zeidler C., Ritter P.R., Tannapfel A., Uhl W. (2010) Determinants of glucose control in patients with chronic pancreatitis. Diabetologia 53, 1062–1069 20217037 10.1007/s00125-010-1705-0

[41] Steinberg W.M., Nauck M.A., Zinman B., Daniels G.H., Bergenstal R.M., Mann J.F. (2014) LEADER 3–lipase and amylase activity in subjects with type 2 diabetes: baseline data from over 9000 subjects in the LEADER Trial. Pancreas 43, 1223–1231 10.1097/MPA.0000000000000229 25275271 PMC4206347

